# Ruptured aneurysm: therapy of abdominal compartment syndrome post EVAR

**DOI:** 10.1186/1471-2318-11-S1-A1

**Published:** 2011-08-24

**Authors:** Vittorio Alberti, Pierluigi Costa, Stefano Fazzini, Eugenia Serrao, Sonia Ronchey, Nicola Mangialardi

**Affiliations:** 1 Unit of Vascular Surgery, San Filippo Neri Hospital, Roma, Italy

## Background

**Figure 1:**
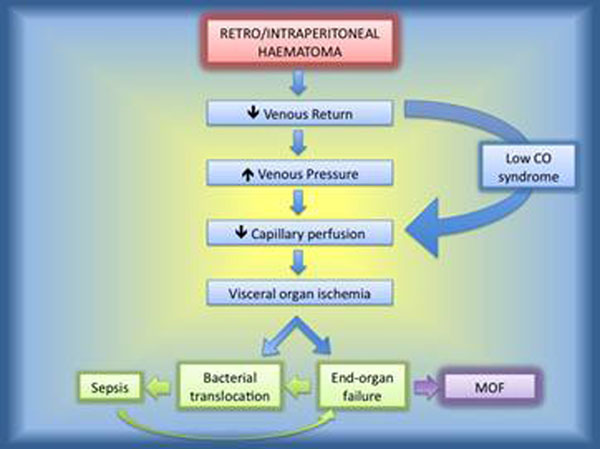
Chain of events triggered by retroperitoneal haematoma.

Endovascular treatment of ruptured abdominal aortic aneurysms (r-EVAR) has the potential to offer improved outcomes. A frequent cause of post-operative mortality following ruptured aortic aneurysm repair is multi-organ failure (MOF) as a consequence of abdominal compartment syndrome (ACS). We reviewed our experience to identify predisposing factors for ACS (Fig. 1) and a way for its treatment.

## Materials and methods

**Figure 2:**
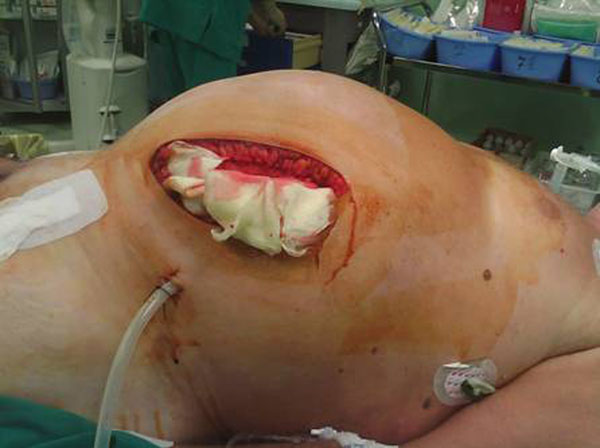
Surgical retroperitoneal access.

From January 2005 to December 2009, 53 patients underwent emergent endovascular repair of r-AAA. We mainly used bifurcated prostheses (44 patients), apart from 5 cases of aorto-uni-iliac device and 4 cases of straight endografts. Nine patients developed ACS and were submitted to abdominal decompression by retroperitoneal surgical drainage (Fig. 2).

## Results

Thirty-day mortality was 22.6% (12/53). Early mortality was recorded in unstable patients only. Stable patients (24) had no mortality in the first 30 days. Among patients who underwent retro-peritoneal drainage, the 30-day mortality rate was 33.3% (3/9). At a median follow up of 34 months (33.8 + 17.0) 3 patients died of aneurysm or procedure related causes.

## Conclusions

One of the priorities in the management of r-EVAR is to prevent and eventually treat the ACS. A surgical evacuation of the retroperitoneal hematoma through extraperitoneal access has considerable advantages, mainly in high risk and older patients. In r-EVAR the particular factor is the retroperitoneal hematoma. Therefore we perform abdominal decompression via retroperitoneal access.
